# Clinical effect evaluation and correlation between preoperative imaging parameters and clinical effect of endoscopic Transforaminal decompression for lumbar spinal stenosis

**DOI:** 10.1186/s12891-020-3076-0

**Published:** 2020-02-03

**Authors:** Lijun Li, Feng Chang, Yong Hai, Jincai Yang, Cheng Xu, Jie Yuan, Jiuqiang Sun, Qinghua Wang, Shengqiang Ding, Xiaowen Yang

**Affiliations:** 1 Department of Orthopedics, Capital Medical University Affiliated Beijing Chaoyang Hospital, Beijing, China; 2 Department of Orthopedics, Shanxi Provincial People’s Hospital, Shanxi, China

**Keywords:** Endoscopic transforaminal discectomy, Lumbar spinal stenosis, Imaging parameters

## Abstract

**Background:**

The objective of this study was to evaluate the clinical effect and correlation between preoperative imaging parameters and the clinical effect of endoscopic transforaminal decompression for lumbar spinal stenosis.

**Methods:**

In this prospective study, 87 patients from Shanxi Province People’s Hospital met the criteria for lumbar spinal stenosis and were recruited from June 2014 to January 2016. These patients underwent endoscopic transforaminal decompression. The clinical symptoms were evaluated by VAS, ODI, and claudication at 3 and 6 months after surgery. The overall clinical efficacy was evaluated using the MacNab score. Yellow ligament thickness and area of the dural sac were examined by MRI. Bony vertebral canal area, real spinal canal area, nerve root canal bony area, nerve root canal real area, distance between the articular joints, and vertebral canal sagittal diameter were examined by CT. The soft tissue invasion ratio of the vertebral canal and the invasion ratio of the nerve root canal were calculated. Correlations between imaging parameters and age, sex, and clinical efficacy were examined.

**Results:**

The MacNab scores were excellent in 47% of cases, good in 34%, generally good in 8%, and poor in 11%. VAS, ODI, and claudication were significantly improved compared with the preoperative values (*P* < 0.01). A significant difference was observed between the 71–81 year age group and the other age groups (*P* < 0.05). There were good correlations between clinical efficacy and vertebral canal sagittal diameter, distance between the articular joints, soft tissue invasion ratio of the vertebral canal, and invasion ratio of the nerve root canal.

**Conclusion:**

Treatment of lumbar spinal stenosis by endoscopic transforaminal decompression can achieve good clinical results. This operation is less effective in patients older than 71 years of age. There were positive correlations between clinical efficacy and the vertebral canal sagittal diameter, the articular joints, soft tissue invasion ratio of the vertebral canal, and invasion ratio of the nerve root canal.

## Background

Percutaneous endoscopic lumbar discectomy (PELD) can be used for the treatment of various types of lumbar intervertebral disc herniation. With the improvements of instruments and equipment, percutaneous spinal endoscopic technology can now be used to treat lumbar spinal stenosis, and satisfactory clinical effects are achieved. The advantages of PELD include its minimal invasiveness (8 mm incision), ability to penetrate physiological channels (intervertebral bore), and minimal tissue destruction. Nevertheless, the treatment of lumbar spinal stenosis with PELD is still being questioned by traditional open surgeons. We currently use percutaneous spinal endoscopic technology to treat lumbar spinal stenosis in some patients, and from our preliminary clinical observations, most postoperative patients achieve satisfactory clinical effects, but the actual clinical curative effects still have to be assessed. Lumbar spinal stenosis is caused by bony stenosis (articular process hyperplasia of cohesion, hyperplasia of vertebral rear osteophyte, and calcified discs) and soft tissue factors (yellow ligament proliferous hypertrophy and uncalcified discs). Therefore, we speculate that the reason for poor efficacy may be that intervertebral endoscopic surgery is better for the relief of soft tissue compression and limited for the treatment of osseous compression. Therefore, the objective of this study was to evaluate the clinical effect and correlation between preoperative imaging parameters and the clinical effect of PELD for lumbar spinal stenosis.

## Methods

### Study design and participants

This was a prospective study of 87 patients with a first diagnosis of lumbar spine stenosis at Shanxi Province People’s Hospital between June 2014 and January 2016.

The inclusion criteria were: 1) 25–85 years of age; 2) unilateral or bilateral symptoms of nerve root with (or without) cauda equina compression; 3) single or double segment stenosis symptoms (patients with three or more stenosed segments undergo open surgery were excluded); 4) intermittent neurogenic claudication; and 5) imaging findings consistent with clinical symptoms in terms of pain location or affected root nerve: i) lateral crypt and/or intervertebral pore stenosis; ii) central vertebral canal and/or mixed spinal stenosis; and iii) failure to 3–6 months of conservative treatment.

The exclusion criteria were: 1) intervertebral instability (in accordance with the White criteria, i.e., over-extension and over-flexion X-ray images in the standing position show that the horizontal displacement of adjacent vertebrae is ≥3 mm or the angle change is ≥15° [1]), osseous infection, mental abnormalities, tumor(s), or communication difficulties; or 2) symptoms of stenosis caused by pure intervertebral disc herniation.

The following rules were applied in the presence of stenosis in more than one segment. 1) If CT and MRI showed two-segment stenosis, and if the clinical symptoms and signs (according to the sensory manifestations and mobility in the innervated areas of the corresponding nerve roots) also met the clinical manifestations of two-segment stenosis, the patients were considered as having two-segment stenosis; they were then included. 2) If CT and MRI showed two-segment stenosis, the clinical symptoms and signs did not show any evident manifestations in the innervated areas of the corresponding nerve roots, and the patient only had the symptom of intermittent neurogenic claudication, but without being able to determine from which segment this symptom was from, the patients were also considered as being two-segment stenosis; they were then included. 3) If CT and MRI showed three or more segment stenosis, but the clinical symptoms and signs showed that they were caused by two-segment stenosis (meaning that the symptoms were restricted to the innervated areas of two segments), the patients were considered as having two-segment stenosis. They were included in the study. 4) If CT and MRI showed three or more segment stenosis, and the clinical symptoms and signs showed that they were caused by three-segment stenosis (meaning that the symptoms were determined as coming from three segments), the patients were considered as having three of more segment stenosis and were excluded. 5) If CT and MRI showed three or more segment stenosis, but the clinical symptoms and signs could not identify the responsible segments, and none of the segments could be ruled out for inducing the disease, the patients were considered with three of more segmental stenosis, and were excluded from the study.

### Surgical methods

According to the patient’s preoperative CT and MRI parameters, and their clinical symptoms, the segment of interest for decompression was identified. A physician with experience in treating lumbar spinal stenosis by PELD performed the surgery routinely and ensured complete decompression during the operation. The operation was performed through the intervertebral foramen (Additional file 1: Figure S1).

### Preoperative and postoperative functional assessment

Clinical symptoms were assessed using the visual analog pain score (VAS) [2, 3], Oswestry Disability Index (ODI) [4, 5], and claudication distance (m). These factors were evaluated independently by two orthopedic surgeons during the preoperative period and at 3 and 6 months postoperatively. The mean value was used as the standard for evaluating preoperative and postoperative clinical symptoms. The overall clinical efficacy was assessed using the MacNab scores [6]. The minimal clinically important difference for ODI is 12.4 [7].

### Preoperative imaging parameter measurement

Patients were evaluated by MRI (Germany Siemens 3.0 T superconducting magnetic resonance instrument) and CT (Germany’s Siemens 64 layer spiral CT machine). The following indicators were measured by two radiologists who were blinded to the patient’s condition. CT and MRI analysis software was used to calculate the mean values. MRI was used to measure the yellow ligament thickness and area of the dural sac (Additional file 1: Figure S2). CT was used to measure the bony vertebral canal area, real spinal canal area, nerve root canal bony area, nerve root canal real area, distance between the articular joints, and vertebral canal sagittal diameter (Additional file 1: Figure S3).

### Definitions

The soft tissue invasion ratio of the vertebral canal and invasion ratio of the nerve root canal were calculated as: the soft tissue invasion ratio of the vertebral canal = (bony vertebral canal area- the real spinal canal area)/bony vertebral canal area; the invasion ratio of the nerve root canal = (nerve root canal bony area - nerve root canal real area)/nerve root canal bony area.

### Statistical analysis

SPSS 22.0 (IBM, Armonk, NY, USA) and GraphPad Prism 6 (GraphPad Software, San Diego CA, USA) were used for statistical analysis. Normally distributed data (according to the Kolmogorov-Smirnov test) are presented as means ± standard deviation, while non-normally distributed data are presented as median (IQR). Categorical variables are presented as frequencies. The Friedman analysis was used to compare efficacy indicators of different time points among multiple groups, and Dunn’s test was used for comparison between groups. The comparisons of sex and age groups were performed using two-way repeated measure ANOVA with the Bonferroni post hoc test. Correlation between sex, age, and CT/MRI parameters and correlation between CT/MRI parameters and therapeutic effects were analyzed by Spearman correlation. Multiple linear regression was used to analyze the association among various indicators. Two-sided *P*-values < 0.05 were considered statistically significant. No multiple testing adjustments were done.

## Results

### Characteristics of the participants

Of the recruited participants, 45 were male (51.7%), and 42 were female (48.3%). The median age was 58 years (range 43–67 years). Table 1 presents the preoperative characteristics of the participants.

**Table 1:** Baseline data and CT/MRI parameters

Variables	All patients (*n* = 87)
Age (years)	58 (43,67)
Male	45 (51.7%)
Bony vertebral canal area by CT measurement (mm^2^)	253.8 ± 52.0
Cross-sectional area of the dural sac by MRI measurement (mm^2^)	82.9 (62.5112.5)
Nerve root canal bony area by CT measurement (mm^2^)	79.0 ± 30.1
Yellow ligament thickness by MRI measurement (mm)	5.2 (4.3,5.6)
Real spinal canal area by CT measurement (mm^2^)	35.3 (28.5,45.2)
Nerve root canal real area by CT measurement (mm^2^)	45.1 (31.3,59.4)
Vertebral canal sagittal diameter by CT measurement (mm)	19.8 (18.1,20.9)
Distance between the articular joints by CT measurement (mm)	16.4 (15.3,18.0)
Soft tissue invasion ratio of the vertebral canal	0.86 (0.82,0.88)
Invasion ratio of the nerve root canal	0.42 ± 0.12

### Evaluation of surgical efficacy

The MacNab score was excellent in 47% of the patients, good in 34%, generally good in 8%, and poor in 11%. The overall optimal rate was 81.1%.

Postoperative VAS, ODI, and claudication at 3 months were significantly improved compared with the preoperative values (*P* < 0.01). Postoperative VAS, ODI, and claudication at 6 months were significantly improved compared with the preoperative values (P < 0.01) (Table 2). These results indicate that PELD was effective in these patients. The changes from baseline in ODI scores are greater than the minimal clinical significant change of 12.4.

**Table 2:** Comparisons of indications about treatment effects among different time points

Variables	Before surgery	3 months after surgery	6 months after surgery	P
VAS	63.4 (57.5,70.6)	11.1 (9.4,15.6)^a^	5.6 (0.0,8.9)^ab^	< 0.001
ODI	57.8 (50.0,66.7)	6.7 (4.4,8.9)^a^	4.0 (2.2,6.7)^ab^	< 0.001
Walking distance (m)	45.5 (16.5205.5)	1500.0 (1500.0,1500.0)^a^	1500.0 (1500.0,1500.0)^a^	< 0.001

a: Compared with the time point before surgery, results showed a significant difference

b: Compared with the time point of 3 months after surgery, results showed a significant difference

**Fig. 1:**
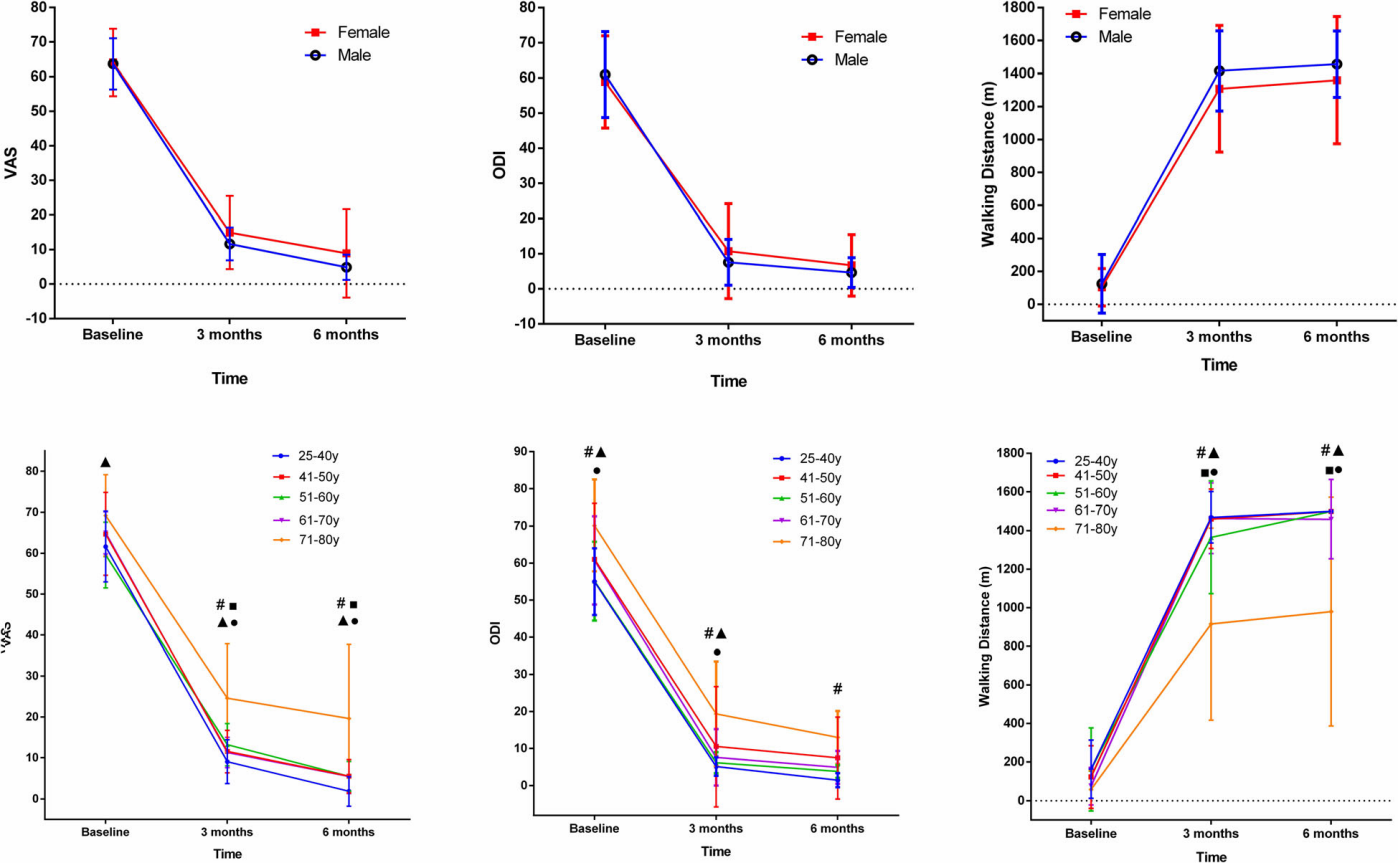
Two-way repeated measure ANOVA by sex stratification. #: 25–40 vs. 71–80, results showed a significant difference. ■: 41–50 vs. 71–80, results showed a significant difference. ▲: 51–60 vs. 71–80, results showed a significant difference. ●: 61–70 vs. 71–80, results showed a significant difference

There was no statistically significant difference between sexes (*P* > 0.05). On the other hand, in the curative effect comparison among the different age groups, a significant difference was observed between the 71–81 year age group and the other age groups (*P* < 0.05). The overall analysis revealed that the 71–81-year group had worse postoperative effects than the other age groups (Fig. 1).

### Correlation of the preoperative imaging parameters with age, sex, and clinical efficacy

There was no significant correlation between most imaging parameters and sex (*P* > 0.05). There was a correlation between the soft tissue invasion ratio of the vertebral canal and the invasion ratio of the nerve root canal with sex (*P* < 0.05) (Table 3).

**Table 3:** Correlation analysis between sex and CT/MRI parameters

	r	P
Bony vertebral canal area by CT measurement	− 0.102	0.349
Cross-sectional area of the dural sac by MRI measurement	−0.607	0.538
Nerve root canal bony area by CT measurement	−0.025	0.820
Yellow ligament thickness by MRI measurement	0.015	0.893
Real spinal canal area by CT measurement	0.188	0.081
Nerve root canal real area by CT measurement	0.080	0.463
Vertebral canal sagittal diameter by CT measurement	−0.170	0.115
Distance between the articular joints by CT measurement	0.047	0.667
Soft tissue invasion ratio of the vertebral canal	−0.211	0.049
Invasion ratio of the nerve root canal	−0.215	0.045

The actual spinal canal area was positively correlated with age (*P* < 0.05). The soft tissue invasion ratio of the vertebral canal and the invasion ratio of the nerve root canal was negatively correlated with age (*P* < 0.05) (Table 4).

**Table 4:** Correlation analysis between age and CT/MRI parameters

	r	P
Bony vertebral canal area by CT measurement	−0.008	0.939
Cross-sectional area of the dural sac by MRI measurement	−0.162	0.133
Nerve root canal bony area by CT measurement	−0.014	0.895
Yellow ligament thickness by MRI measurement	−0.010	0.927
Real spinal canal area by CT measurement	0.223	0.038
Nerve root canal real area by CT measurement	0.125	0.249
Vertebral canal sagittal diameter by CT measurement	−0.041	0.707
Distance between the articular joints by CT measurement	0.007	0.948
Soft tissue invasion ratio of the vertebral canal	−0.228	0.033
Invasion ratio of the nerve root canal	−0.360	0.001

A good correlation was observed between VAS and articular joints by CT, soft tissue invasion ratio of the vertebral canal, and invasion ratio of the nerve root canal (*P* < 0.05); ODI and articular joints by CT, soft tissue invasion ratio of the vertebral canal, and invasion ratio of the nerve root canal (*P* < 0.05); and walking distance and articular joints by CT, soft tissue invasion ratio of the vertebral canal, and invasion ratio of the nerve root canal (*P* < 0.05) (Table 5).

**Table 5:** Correlation analysis between CT/MRI parameters and therapeutic effects

	VAS		ODI		Walking distance	
	r	P	r	P	r	P
Bony vertebral canal area by CT measurement	0.166	0.124	0.167	0.122	0.136	0.210
Cross-sectional area of the dural sac by MRI measurement	0.112	0.304	0.024	0.826	0.195	0.071
Nerve root canal bony area by CT measurement	−0.119	0.272	0.035	0.750	0.001	0.992
Yellow ligament thickness by MRI measurement	0.162	0.133	0.012	0.909	0.122	0.260
Real spinal canal area by CT measurement	−0.041	0.705	−0.102	0.348	0.044	0.687
Nerve root canal real area by CT measurement	−0.179	0.098	−0.103	0.344	0.008	0.943
Vertebral canal sagittal diameter by CT measurement	0.139	0.198	0.050	0.643	0.055	0.613
Distance between the articular joints by CT measurement	0.366	< 0.001	0.222	0.039	0.377	< 0.001
Soft tissue invasion ratio of the vertebral canal	0.228	0.021	0.213	0.047	0.261	0.024
Invasion ratio of the nerve root canal	0.262	0.014	0.290	0.006	0.350	0.001

### Multiple linear regression of VAS, ODI and walking distance in 6 months

There was a significant correlation between the soft tissue invasion ratio of the vertebral canal, invasion ratio of the nerve root canal, actual spinal canal area, and nerve root canal real area with VAS (*P* < 0.05) (Table 6).

**Table 6:** Linear regression of different indicators (multiple linear regression)

Parameters	Non-standardized β	Standardized β	P
VAS (6 months)			
Soft tissue invasion ratio of the vertebral canal	71.05	0.542	< 0.001
Invasion ratio of the nerve root canal	28.06	0.347	0.015
Real spinal canal area by CT measurement	0.3	0.023	0.011
Nerve root canal real area by CT measurement	−0.08	0.01	0.034
ODI (6 months)			
Soft tissue invasion ratio of the vertebral canal	31.592	0.375	< 0.001
Walking distance (6 months)			
Soft tissue invasion ratio of the vertebral canal	2296.6	0.435	< 0.001
Invasion ratio of the nerve root canal	635.4	0.376	0.028
Real spinal canal area by CT measurement	6.21	0.025	0.04

There was a significant correlation between the distance between the articular joints and the soft tissue invasion ratio of the vertebral canal with ODI (*P* < 0.01) (Table 6).

There was a significant correlation between the distance between the articular joints, soft tissue invasion ratio of the vertebral canal, invasion ratio of the nerve root canal, and real spinal canal area with claudication distance (P < 0.05) (Table 6).

These results indicate that imaging parameters are independently associated with the clinical outcomes after PELD for lumbar segment spinal stenosis.

## Discussion

There is presently no uniform standard regarding the surgical indications for the treatment of lumbar spinal stenosis, and there are different opinions regarding the treatment of lumbar spinal stenosis with PELD. Polikandriotis et al. [8] examined bleeding, time, pain, and functional improvement before and after PELD compared with conventional surgery, and consider that PELD through the laminae or foramen is safe and effective for the treatment of lumbar spinal stenosis. Polikandriotis et al. [8] microscopically treated lumbar spinal stenosis disease through the intervertebral foramen and noted that the operation time for this type of surgery was short, hemorrhage was minimal, and operative and postoperative complications were slightly more frequent than those with traditional surgery. Thus, these authors concluded that this approach is safe and effective for lumbar spinal stenosis. Ahn [9] summarized some of the surgical techniques for the treatment of lumbar vertebral stenosis in different parts of the body through the percutaneous intervertebral foramen and discussed the advantages and disadvantages of selecting the laminae or foramen in different parts of the lumbar vertebra. Lawrence et al. [10] summarized the relevant information on the treatment of lumbar spinal stenosis using percutaneous intervertebral foramen in multiple hospitals, and the results showed that all patients had good clinical effects and no significant differences compared with conventional surgery. These authors also compared the advantages of the technique in regard to safety and treatment costs. Finally, these authors concluded that percutaneous intervertebral foramen mirror technology could effectively solve most cases of lumbar spinal stenosis, and even if it is difficult to reach these areas during surgery, it may not be sufficient to relieve the stress. If blind decompression has the potential to increase the risk of surgery, the doctor should weigh its advantages and disadvantages. Xu [11] believed that PELD could also be used to treat lumbar spinal stenosis disease by expanding the intervertebral foramen and removing the yellow lateral ligament during the treatment of lateral stenosis. This author also noted the relatively limited scope of the operation; for severe hyperplasia and serious intervertebral foramen stenosis, it is difficult to enter the intervertebral pore, which requires a drill and bone chisel along with longer operation time. Nevertheless, the surgical indications for lumbar spinal stenosis surgery PELD and whether they are the same as for traditional open surgery remain to be defined.

Imaging measurement of lumbar spinal stenosis is an auxiliary and indispensable examination that can be performed for diagnosis. Zhang et al. [12] accurately delineated the boundary of the area and measured the area, angle, and segment length of the lesion using measurement software. Liu [13] measured the bones of 100 corpses, and the diameter of the spinal canal in L4 was 15.31 ± 2.23 mm, and that in L5 was 15.98 ± 2.58 mm. Guo [14] et al. measured the diameter of the L5 vertebral canal to be 16.61 ± 2.42 mm. In the present study, the canal in L4 was 15.44 ± 3.06 mm, and that in L5 was 16.18 ± 2.29 mm. The results showed that measurement of the diameter of the vertebral canal was very similar to that of previous studies, indicating that the CT images are close to the physical specimen measurement.

We treated 87 cases of lumbar spinal stenosis with percutaneous endoscopic technology. The results showed that the VAS, ODI, and claudication distance were significantly improved compared with baseline. A significant difference in efficacy was observed between the 71–81 year age group and the other age groups. The actual spinal canal area was positively correlated with age. The soft tissue invasion ratio of the vertebral canal and invasion ratio of the nerve root canal were negatively correlated with age. There was a correlation between the soft tissue invasion ratio of the vertebral canal and the invasion ratio of the nerve root canal with sex. Correlation analysis of the imaging parameters and effect revealed a good correlation between the clinical efficacy and vertebral canal sagittal diameter, distance between the articular joints, soft tissue invasion ratio of the vertebral canal, and invasion ratio of the nerve root canal. These measurements were positively correlated with VAS, ODI, and claudication distance.

## Conclusion

Based on the above results, the following conclusions can be drawn. The treatment of lumbar spinal stenosis by endoscopic transforaminal decompression can achieve good clinical results, but the operation could be less effective in patients > 71 years of age. Whether age > 71 is a contraindication of treatment of lumbar spinal stenosis with percutaneous endoscopic technology remains to be confirmed. There was a positive correlation between the vertebral canal sagittal diameter, distance between the articular joints, soft tissue invasion ratio of the vertebral canal, and invasion ratio of the nerve root canal with the clinical effect. This is in line with the characteristics of the intervertebral foramen mirror because of the greater influence factors, such as age.

In conclusion, although percutaneous endoscopic decompression surgery can have good clinical efficacy and unique minimally invasive advantages in the treatment of degeneration of spinal canal stenosis [15–18], the surgical indications are dependent on a good preoperative evaluation because the intervertebral foramen is still a greater challenge than lumbar intervertebral disc herniation. It is also important to focus on the complications of surgery such as dural sac and nerve injury [19, 20], as well as radiation exposure [21]. Due to the limited amount of clinical data collection and limited follow-up time, the results of this study have a clear guiding significance for the clinical application of intervertebral endoscopy in the treatment of lumbar spinal stenosis, which requires further study and exploration.

## Supplementary information

**Additional file 1: Figure S1.** Surgical methods. **Figure S2.** Preoperative MRI measurement. **Figure S3.** Preoperative CT measurement.

## Data Availability

The datasets used and/or analyzed during the current study are available from the corresponding author on reasonable request.

## Abbreviations

ODI: Oswestry Disability Index
PELD: percutaneous endoscopic lumbar discectomy;
VAS: Visual Analog pain score;
