# Supplementary material for: Clinical effect evaluation and correlation between preoperative imaging parameters and clinical effect of endoscopic Transforaminal decompression for lumbar spinal stenosis
Source: BMC Musculoskelet Disord. 2020 Feb 3;21:68. doi: 10.1186/s12891-020-3076-0 (PMC6998066; doi:10.1186/s12891-020-3076-0)
Supplement: Supplementary file 1 — Additional file 1: Figure S1. Surgical methods. Figure S2. Preoperative MRI measurement. Figure S3. Preoperative CT measurement. [file 12891_2020_3076_MOESM1_ESM.doc]

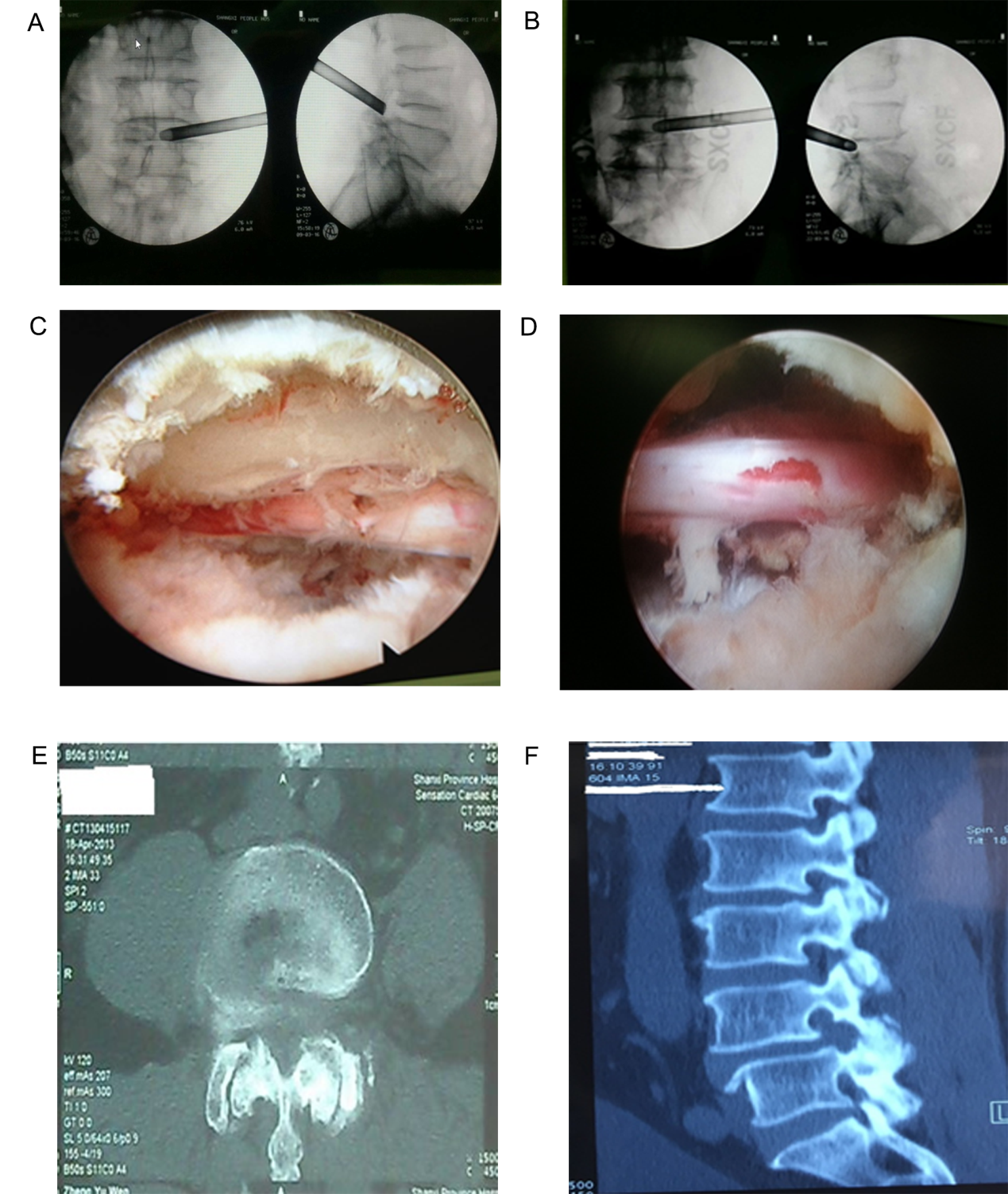


**Figure S1.** Surgical methods. (A) The passageway can be placed in the rear of the dura capsule to reach the midline; (B) The passageway can be placed in front of the dura capsule to reach the midline or to the opposite side; (C) Pre-decompression microscopic display; (D) Post-decompression microscopic display; (E) CT coronary view shows the decompression of the nerve root canal and the central vertebral canal; (F) CT sagittal view shows the decompression of the root canal.


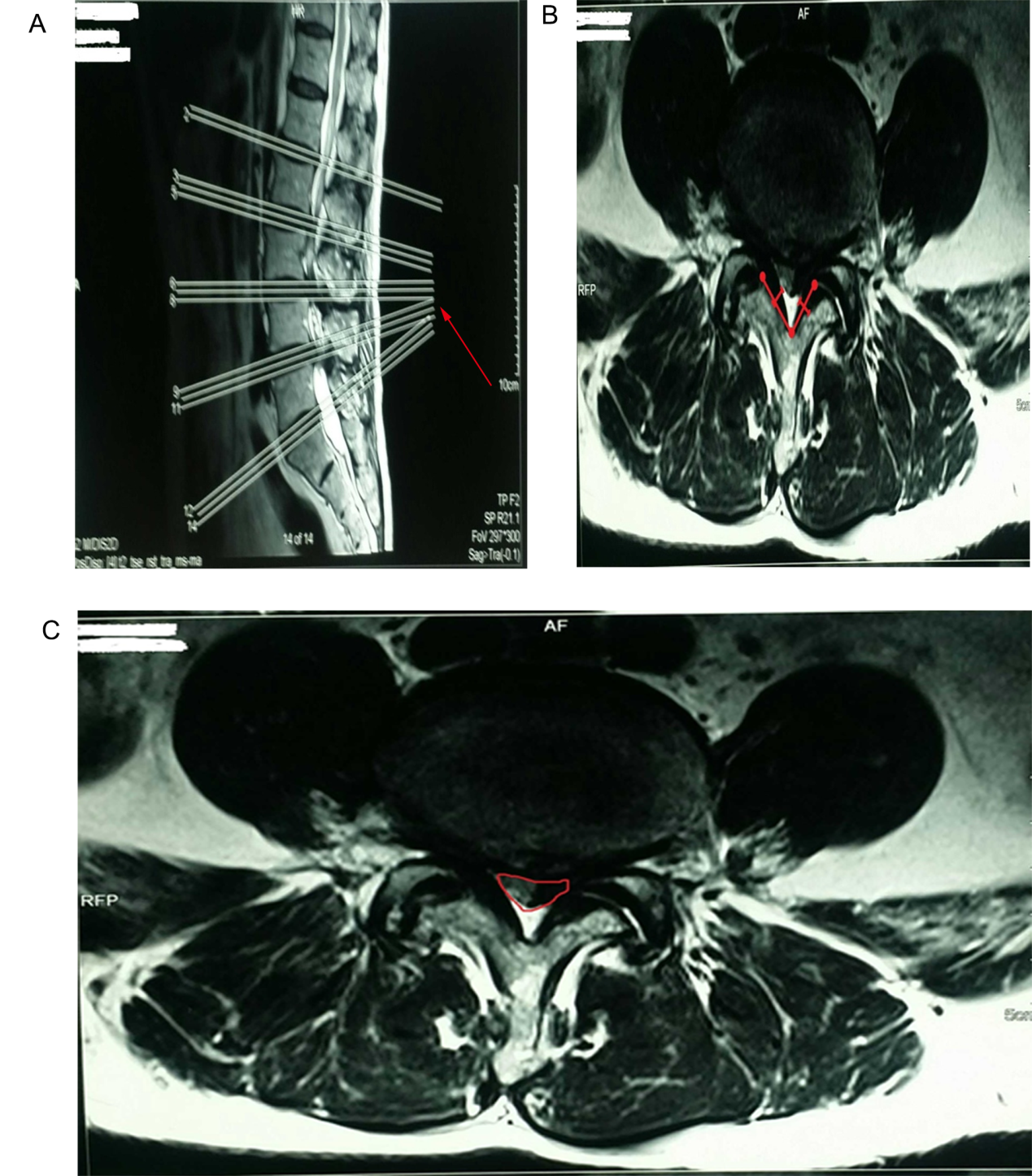


**Figure S2.** Preoperative MRI measurement. (A) Sagittal view of the cross-sectional area of the vertebral canal by MRI. The arrow shows the scanning results of the middle of the interstitial three layers; (B) Thickness of the yellow ligaments measured by MRI. The measured region is the midpoint of the connection line between the vertex of the upper joint and the joint of the lamina. The mean value was calculated; (C) Transverse section of the preoperative dural sac measured by MRI.


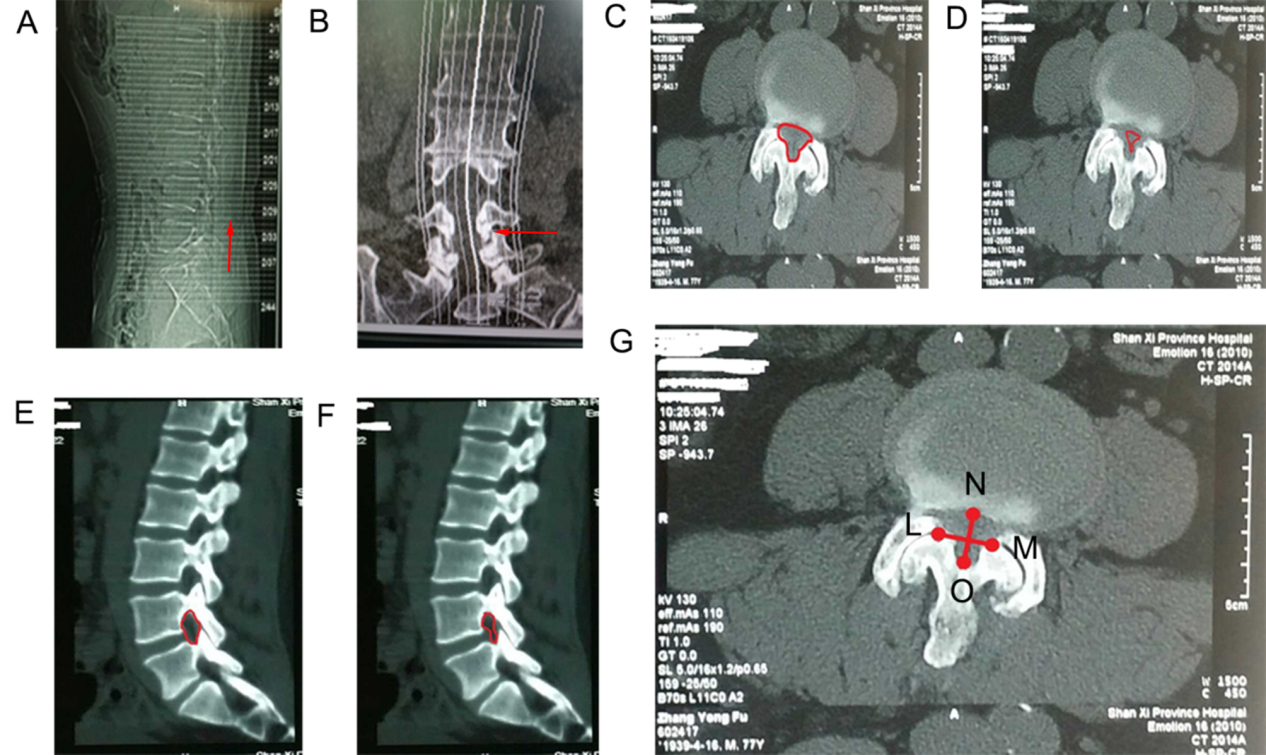


**Figure S3.** Preoperative CT measurement. (A) Sagittal view of the cross-sectional area of the vertebral canal measured by CT. The arrow shows the results of the middle of the interstitial three layers; (B) Area of the intervertebral pore measured by CT. The arrow shows the results of the joint interarticulation; (C) Bony vertebral canal area measured by CT; (D) Real spinal canal area measured by CT; (E) Nerve root canal bony area measured by CT; (F) Nerve root canal real area measured by CT; (G) Distance between the articular joints measured by CT. The sagittal diameter of the vertebral canal was measured by CT. The distance between the midpoint of the posterior margin of the vertebral body and the interconnecting point of both sides of the vertebral plate was measured.
